# Supplementary material for: Help seeking for intimate partner violence in a resource-constrained setting: A latent class analysis of the Nigerian demographic health survey dataset
Source: PLoS One. 2025 Oct 22;20(10):e0334905. doi: 10.1371/journal.pone.0334905 (PMC12543103; doi:10.1371/journal.pone.0334905)
Supplement: S1 Table — Complete version of Table 1. (DOCX) [file pone.0334905.s001.docx]

**S1 Table:** **Background characteristics of women who experienced IPV compared with those who did not (n = 8,910)***

| **Characteristic** | **Total Study Population**  **(n = 8,190)** | **Women who experienced IPV**  **(n = 3,054)** | **Women who did not experience IPV**  **(n = 5.136)** | **p value** |
| --- | --- | --- | --- | --- |
| **Age (years)^&^** | 32.2 (11.33) | 31.9 (13.53) | 32.8 (19.14) | = 4.7×10^−4^ |
| **Number of Children^&^** | 3.5 (3.78) | 3.5 (4.00) | 3.4 (6.50) | 0.25 |
| **Region^#^** |  |  |  |  |
| North Central | 14.0 | 15.7 | 10.8 | < 2.2e^-16^ |
| Northeast | 15.4 | 18.7 | 9.1 |  |
| North West | 29.7 | 30.8 | 27.6 |  |
| South East | 11.5 | 11.3 | 12.0 |  |
| South South | 10.8 | 12.1 | 8.4 |  |
| South West | 18.5 | 11.4 | 32.1 |  |
| **Place of Residence^#^** |  |  |  |  |
| Urban | 43.3 | 39.6 | 50.7 | = 3.995e^-11^ |
| Rural | 56.7 | 60.4 | 49.3 |  |
| **Highest Educational Level^#^** |  |  |  |  |
| No education | 41.1 | 43.0 | 37.6 | = 1.503e^-08^ |
| Primary | 16.6 | 17.2 | 15.3 |  |
| Secondary | 32.8 | 32.0 | 34.3 |  |
| Higher | 9.5 | 7.8 | 12.8 |  |
| **Religion^#^** |  |  |  |  |
| Catholic | 9.8 | 9.8 | 9.7 |  |
| Other Christian | 34.6 | 34.2 | 35.3 |  |
| Islam | 55.1 | 55.5 | 54.4 |  |
| Traditionalist | 0.4 | 0.4 | 0.4 |  |
| Other | 0.1 | 0.1 | 0.2 |  |
| **Wealth Index^#^** |  |  |  |  |
| Poorest | 18.6 | 20.0 | 15.7 | = 1.107e^-12^ |
| Poorer | 20.1 | 21.1 | 18.1 |  |
| Middle | 20.6 | 21.9 | 18.1 |  |
| Richer | 20.5 | 20.0 | 21.4 |  |
| Richest | 20.3 | 16.9 | 26.7 |  |
| **Visit to Health Facility in last 12 months^#^** |  |  |  |  |
| No | 53.0 | 50.5 | 57.7 | = 4.249e^-06^ |
| Yes | 47.0 | 49.5 | 42.3 |  |
| **Marital Status^#^** |  |  |  |  |
| Married | 90.3 | 89.9 | 91.2 | = 1.935e^-09^ |
| Living with Partner | 3.5 | 3.9 | 2.8 |  |
| Widowed | 3.2 | 2.4 | 4.5 |  |
| Divorced | 1.4 | 1.8 | 0.6 |  |
| Separated | 1.6 | 2.0 | 0.9 |  |
| **Woman’s attitude to IPV^#^** |  |  |  |  |
| Non-Accepting | 71.0 | 66.6 | 79.6 | < 2.2e^-16^ |
| Accepting | 29.0 | 33.4 | 20.4 |  |
| **Intergenerational History of IPV^#^** |  |  |  |  |
| No | 85.0 | 81.4 | 92.0 | < 2.2e^-16^ |
| Yes | 9.9 | 12.5 | 4.8 |  |
| Don’t Know | 5.1 | 6.1 | 3.2 |  |
| ***Number of Other Wives^&^** | 0.71 (6.02) | 0.8 (7.58) | 0.6 (8.95) |  |
| ***Highest Educational Level of Husband/Partner^#^** |  |  |  |  |
| No education | 31.3 | 32.2 | 29.7 | = 0.009 |
| Primary | 14.8 | 15.3 | 13.8 |  |
| Secondary | 36.2 | 35.3 | 38.1 |  |
| Higher | 16.1 | 15.4 | 17.5 |  |
| Don’t Know | 1.5 | 1.8 | 0.9 |  |

^&^Mean and Standard Deviation

^#^Percentages (these may not add up to 100 due to rounding)

*Complete version of Table 1
