# Supplementary material for: Help seeking for intimate partner violence in a resource-constrained setting: A latent class analysis of the Nigerian demographic health survey dataset
Source: PLoS One. 2025 Oct 22;20(10):e0334905. doi: 10.1371/journal.pone.0334905 (PMC12543103; doi:10.1371/journal.pone.0334905)
Supplement: Attachment — Submitted filename: PONE-D-24-53009_reviewer.pdf [file pone.0334905.s004.pdf]

PLOS ONE

Help seeking for intimate partner violence in a resource-constrained setting: A latent class analysis of the Nigerian demographic health survey dataset

--Manuscript Draft--

**Manuscript Number:**	PONE-D-24-53009
**Article Type:**	Research Article
**Full Title:**	Help seeking for intimate partner violence in a resource-constrained setting: A latent class analysis of the Nigerian demographic health survey dataset
**Short Title:**	Latent classes of help seeking behaviour
**Corresponding Author:**	Omolola Titilayo Alade, BDS (Ib), MIPH (Syd)<br>McMaster University Faculty of Health Sciences<br>Hamilton, Ontario CANADA
**Keywords:**	Intimate Partner Violence, Help Seeking, Latent Variables, Low-and-middle-income country, Nigeria
**Abstract:**	Intimate partner violence (IPV) disproportionately affects women and is further exacerbated in resource-constrained settings by limited sources of help. We therefore aimed to conduct a latent class analysis of help seeking behaviour among women experiencing IPV in Nigeria, a resource-constrained setting. A latent class analysis of the 2018 Nigeria Demographic Health Survey (DHS) data was conducted. Help seeking was defined by the latent class indicators of the places or people where women sought help. The data were analysed in MPlus version 8.10 and survey sampling weights were applied. The relative fit of the models was compared using Bayesian Information Criterion (BIC), Adjusted BIC (ABIC), Lo-Mendell-Rubin Likelihood Ratio Test (LMR) p-values, and entropy values. Of the 3,054 women who experienced physical or sexual violence, 1,041 (33%) women reported seeking for help and a four-class model of help seeking behaviour (BIC = 3910.80, ABIC = 383770, LMR p-value = 00002, and entropy value = 092) was described: Class I (Own Family; 49%), Class II (Everywhere; 18%), Class III (Predominantly Formal; 5%), and Class IV (Predominantly Partner's Family; 28%). The factors significantly associated with these latent classes were region of the country, previous sexual violence, and the woman's highest level of education. Women evinced a high reliance on informal sources for help. However, women with a history of sexual violence were most likely to access formal sources of help. Both informal and formal systems need to be strengthened for help seeking for IPV.
**Order of Authors:**	Omolola Titilayo Alade, BDS (Ib), MIPH (Syd)
	Forough Farrokhyar, PhD
	Sheila Ann Sprague, PhD
	Anita Acai, PhD
	Mohit Bhandari, PhD
**Additional Information:**	
**Question**	**Response**
**Financial Disclosure**<br><br>Enter a financial disclosure statement that describes the sources of funding for the work included in this submission. Review the submission guidelines for detailed requirements. View published research articles from *PLOS ONE* for specific examples.	The author(s) received no specific funding for this work.

Powered by Editorial Manager® and ProduXion Manager® from Aries Systems Corporation

This statement is required for submission and **will appear in the published article** if the submission is accepted. Please make sure it is accurate.<br><br>**Funded studies**<br>Enter a statement with the following details:<br>• Initials of the authors who received each award<br>• Grant numbers awarded to each author<br>• The full name of each funder<br>• URL of each funder website<br>• Did the sponsors or funders play any role in the study design, data collection and analysis, decision to publish, or preparation of the manuscript?<br><br>Did you receive funding for this work?	
**Competing Interests**<br><br>Use the instructions below to enter a competing interest statement for this submission. On behalf of all authors, disclose any competing interests that could be perceived to bias this work—acknowledging all financial support and any other relevant financial or non-financial competing interests.<br><br>This statement is required for submission and **will appear in the published article** if the submission is accepted. Please make sure it is accurate and that any funding sources listed in your Funding Information later in the submission form are also declared in your Financial Disclosure statement.<br><br>View published research articles from *PLOS ONE* for specific examples.	The authors have declared that no competing interests exist.

**NO authors have competing interests**<br><br>Enter: *The authors have declared that no competing interests exist.*<br><br>**Authors with competing interests**<br><br>Enter competing interest details beginning with this statement:<br><br>*I have read the journal's policy and the authors of this manuscript have the following competing interests: [insert competing interests here]*<br><br>* typeset	
**Ethics Statement**<br><br>Enter an ethics statement for this submission. This statement is required if the study involved:<br><br>• Human participants<br>• Human specimens or tissue<br>• Vertebrate animals or cephalopods<br>• Vertebrate embryos or tissues<br>• Field research<br><br>Write "N/A" if the submission does not require an ethics statement.<br><br>General guidance is provided below. Consult the submission guidelines for detailed instructions. **Make sure that all information entered here is included in the Methods section of the manuscript.**	Permission to use the publicly available dataset in this analysis was requested and granted by the demographic and health survey (DHS) program (https://dhsprogram.com/Data/). The DHS Program and the National Health Research Ethics Committee of Nigeria reviewed and approved the research protocol. Each respondent was read an informed consent statement and could verbally accept or decline to participate with no consequences. Parents or guardians provided consent for adolescents. The consent decision was witnessed and documented on the data collection forms by the interviewers. The data were only made publicly available after they were fully anonymized. The Hamilton integrated research ethics board of McMaster University further gave ethical approval for the study (Project ID: 17782).

**Format for specific study types**<br><br>**Human Subject Research (involving human participants and/or tissue)**<br>• Give the name of the institutional review board or ethics committee that approved the study<br>• Include the approval number and/or a statement indicating approval of this research<br>• Indicate the form of consent obtained (written/oral) or the reason that consent was not obtained (e.g. the data were analyzed anonymously)<br><br>**Animal Research (involving vertebrate animals, embryos or tissues)**<br>• Provide the name of the Institutional Animal Care and Use Committee (IACUC) or other relevant ethics board that reviewed the study protocol, and indicate whether they approved this research or granted a formal waiver of ethical approval<br>• Include an approval number if one was obtained<br>• If the study involved *non-human primates*, add *additional details* about animal welfare and steps taken to ameliorate suffering<br>• If anesthesia, euthanasia, or any kind of animal sacrifice is part of the study, include briefly which substances and/or methods were applied<br><br>**Field Research**<br><br>Include the following details if this study involves the collection of plant, animal, or other materials from a natural setting:<br>• Field permit number<br>• Name of the institution or relevant body that granted permission	
**Data Availability**<br><br>Authors are required to make all data underlying the findings described fully available, without restriction, and from the time of publication. PLOS allows rare exceptions to address legal and ethical concerns. See the PLOS Data Policy and FAQ for detailed information.	No - some restrictions will apply

Powered by Editorial Manager® and ProduXion Manager® from Aries Systems Corporation

A Data Availability Statement describing where the data can be found is required at submission. Your answers to this question constitute the Data Availability Statement and **will be published in the article**, if accepted.<br><br>**Important:** Stating 'data available on request from the author' is not sufficient. If your data are only available upon request, select 'No' for the first question and explain your exceptional situation in the text box.<br><br>Do the authors confirm that all data underlying the findings described in their manuscript are fully available without restriction?	
**Describe where the data may be found in full sentences. If you are copying our sample text, replace any instances of XXX with the appropriate details.**<br><br>• If the data are **held or will be held in a public repository**, include URLs, accession numbers or DOIs. If this information will only be available after acceptance, indicate this by ticking the box below. For example: *All XXX files are available from the XXX database (accession number(s) XXX, XXX.)*.<br>• If the data are all contained **within the manuscript and/or Supporting Information files**, enter the following: *All relevant data are within the manuscript and its Supporting Information files.*<br>• If neither of these applies but you are able to provide **details of access elsewhere**, with or without limitations, please do so. For example:<br><br>*Data cannot be shared publicly because of [XXX]. Data are available from the XXX Institutional Data Access / Ethics Committee (contact via XXX) for researchers who meet the criteria for access to confidential data.*<br><br>*The data underlying the results presented in the study are available from (include the name of the third party*	The data underlying the results presented in the study are available from https://dhsprogram.com/data/available-datasets.cfm.

and contact information or URL).<br>• This text is appropriate if the data are owned by a third party and authors do not have permission to share the data.<br><br>* typeset	
Additional data availability information:	

Manuscript
Click here to access/download;Manuscript;Manuscript_LCA_IPV-1.docx

Help Seeking for Intimate Partner Violence in a Resource-Constrained Setting: A Latent Class Analysis of the Nigerian Demographic Health Survey Dataset

Latent classes of help seeking behaviour

Omolola Titilayo Alade[1*], Forough Farrokhyar[1,2,4], Sheila Sprague[2,4], Anita Acai[3,4], Mohit Bhandari[1,2,4]

[1]Global Health Program, Faculty of Health Sciences, McMaster University, Hamilton, Ontario, Canada

[2]Department of Surgery, Faculty of Health Sciences, McMaster University, Hamilton, Ontario, Canada

[3]Department of Psychiatry and Behavioural Neurosciences, McMaster University, Hamilton, Ontario, Canada

[4]Department of Health Research Methods, Evidence and Impact, McMaster University, Hamilton, Ontario, Canada

*Corresponding Author
E-mail: aladeo1@mcmaster.ca (OTA)

Abstract

Intimate partner violence (IPV) disproportionately affects women and is further exacerbated in resource-constrained settings by limited sources of help. We therefore aimed to conduct a latent class analysis of help seeking behaviour among women experiencing IPV in Nigeria, a resource-constrained setting. A latent class analysis of the 2018 Nigeria Demographic Health Survey (DHS) data was conducted. Help seeking was defined by the latent class indicators of the places or people where women sought help. The data were analysed in MPlus version 8.10 and survey sampling weights were applied. The relative fit of the models was compared using Bayesian Information Criterion (BIC), Adjusted BIC (ABIC), Lo-Mendell-Rubin Likelihood Ratio Test (LMR) *p*-values, and entropy values. Of the 3,054 women who experienced physical or sexual violence, 1,041 (33%) women reported seeking for help and a four-class model of help seeking behaviour (BIC = 3910.80, ABIC = 3837·70, LMR *p*-value = 0·0002, and entropy value = 0·92) was described: Class I (Own Family; 49%), Class II (Everywhere; 18%), Class III (Predominantly Formal; 5%), and Class IV (Predominantly Partner's Family; 28%). The factors significantly associated with these latent classes were region of the country, previous sexual violence, and the woman's highest level of education. Women evinced a high reliance on informal sources for help. However, women with a history of sexual violence were most likely to access formal sources of help. Both informal and formal systems need to be strengthened for help seeking for IPV.

Introduction

Intimate partner violence (IPV) is a devastating experience and one of the ways women experiencing it respond is to actively reach out for assistance. This is referred to as help seeking. Help seeking is an important coping strategy in IPV but has a reported average prevalence of 35% in low- and middle-income countries (LMIC).[1] This prevalence was measured in 31 LMICs and ranged from 18% in Mali to 61% in Sierra Leone.[1] A study in Nigeria found that only about 40% of women experiencing IPV sought help to stop the perpetrator from hurting them again. [2] The same study further found that help seeking behaviour varied widely across the states in Nigeria, ranging from 12% to 65%.

The predictors of help seeking behaviour have been categorized as individual, state, and national factors.[2] Individual factors include attitudes towards IPV, empowerment of the woman, as well as the severity and type of violence. State and national factors include human development indices derived from a composite of literacy rates, per capita gross domestic product, life expectancy, and legal policies on IPV such as the definition of rape and state interference with domestic issues. Another study in Nigeria specifically highlighted the type and severity of IPV as predictors of help seeking.[3] They found that women experiencing physical violence were more likely to seek help than those experiencing sexual violence. This was attributed to the culture of silence and stigma associated with sexual acts. They further observed that severe IPV was more likely to result in help seeking.

Help seeking for IPV may progress from individual strategies (behaviour change, negotiating, and resisting) to informal sources (family, friends and neighbours) and finally formal sources (shelters, police, legal, and medical services).[4, 5] This progression is influenced by a complex interplay of individual and contextual factors including the type and severity of the IPV.[4] In high-income countries, help for women experiencing violence spans this spectrum and formal services for women experiencing IPV are available.[4] On the other hand, support

systems for women experiencing IPV in resource-constrained settings are much less structured and largely rely on informal sources.[6] In Nigeria, women experiencing IPV sought help from their own family (22%), partner's family (11%), neighbours and friends (3%) and formal institutions (i.e., healthcare institutions, legal services, social organizations, and police services; 3%).[7]

Latent classes of help seeking have been described to elucidate how women use sources of help for IPV in high income settings. A Canadian study identified the classes as Minimal or No Help Seeking, Seeking Help from Family and Friends Only, and Substantial Help Seeking.[8] A study conducted in Israel described the latent classes as Substantial Use of All Services, Welfare and Criminal System Use, and Minimal Use of All Services.[9] In the United States, the clusters found were Broadly Engages Formal and Informal Networks, Primarily Engages Informal Networks, and Broadly Engages Networks But Avoids Legal Systems.[10] Another study among non-help seeking women in the United States identified three clusters of safety strategies: Exploring Safety Options, Avoiding the Justice System, and Trying Everything.[11]

The latent classes of help seeking for IPV in resource-constrained settings remains unexplored. It is important to fill this research gap and direct targeted interventions to improve help seeking in these settings. Therefore, this study aimed to conduct a latent class analysis (LCA) of help seeking behaviour among women experiencing IPV using data from the most recent Nigerian Demographic Health Survey, with Nigeria being an example of a resource-constrained setting.

Materials and methods

This was an LCA of the 2018 Nigeria Demographic Health Survey (DHS) data, the most recently available survey. The DHS program is an international collaborative effort primarily funded by the United States Agency for International Development (USAID) to conduct nationally representative health surveys in more than 90 low- and middle-income countries for about four decades.[7] In Nigeria, these surveys are implemented by the National Population Commission of Nigeria and were first conducted in 1990.[7] The survey was implemented using computer-assisted personal interviewing and data collection took place from 14 August to 29 December 2018. The dataset was downloaded for analysis on 16 September 2023.

Study Location

Nigeria is a country in West Africa with an estimated population of 200,000,000 people.[12] It is made up of six geo-political zones further divided into 36 states and the Federal Capital Territory, Abuja.[7] These states are further divided into Local Government Areas (LGAs) to total 774 LGAs. LGAs are further divided into enumerations areas (EAs) for a total of 662,529 EAs with an average of 856 EAs per LGA.

Sampling Strategy

A stratified, two-stage cluster design was used in the DHS survey. EAs were stratified into urban and rural areas as the sampling units for the first stage. At this first stage, 1,400 EAs were randomly selected. The second stage was a complete listing of households in the 1,400 EAs, out of which 41,688 households were randomly selected. Of these selected households, 40,666 were occupied and people in 40,427 of these were successfully interviewed, yielding a response rate of 99%.

The target groups in the households were women aged 15-49 years and men aged 15-59 years. The men's survey was conducted in one-third of the sample households, and included all men aged 15-59 years present in these households. In this subsample of households where men were

interviewed, one eligible woman in each household was randomly selected to be asked additional questions about domestic violence. In total, 10,678 women were surveyed about their experiences with domestic violence. Only 8,910 of these 10,678 women (who were ever married or ever had a partner) were asked questions on IPV and 3,054 women reported that they had experienced physical or sexual violence from a partner, even though the definition of IPV in this survey included emotional abuse and control issues as further described below. Only these 3,054 women who reported that they experienced physical and/or sexual IPV were asked if they sought help for IPV, of which 1,041 women reported that they had (Fig 1).

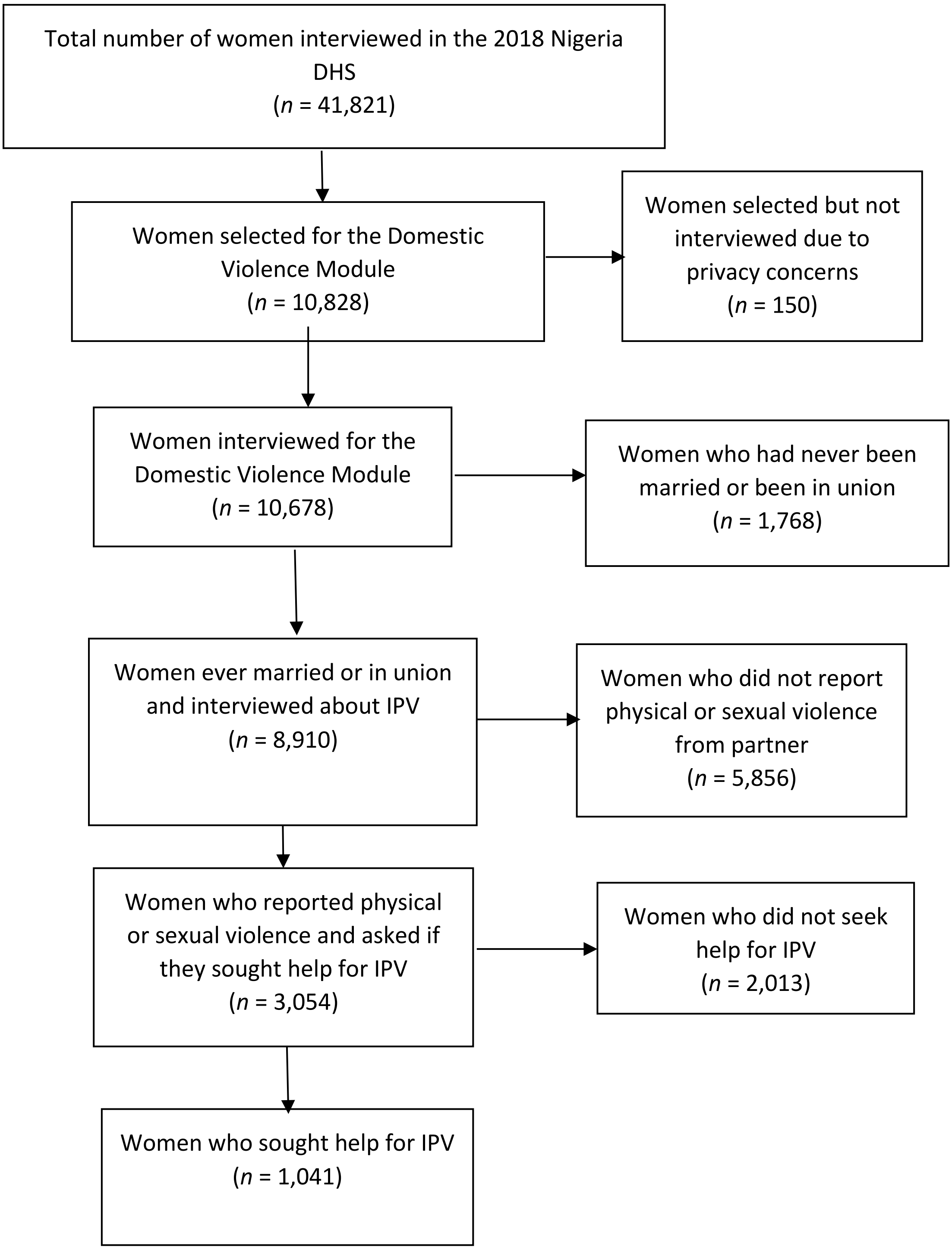

Fig 1. Flow diagram of women included in the LCA

IPV – Intimate partner violence

Theoretical Framework

The current study was based on the theoretical framework of help seeking behaviour in IPV described by Liang et al. [5] This framework outlines individual, interpersonal, and socio-cultural influences on women's help seeking attitudes as they move non-linearly from recognising intimate violence as a problem to taking a decision to seek help and choosing a source of help. The factors selected were limited by availability of data in the DHS dataset. The individual influences on women's help seeking for IPV were assessed by personal characteristics including age, presence or absence of children, level of education, intergenerational history of IPV, attitude towards IPV, and type and severity of IPV. Interpersonal factors are those factors which influence the relationship with an intimate partner, and they include the presence or absence of other wives or partners in the relationship, the woman's marital status, and family connections. Socio-cultural factors are external to the relationship but may shape help seeking for IPV and were assessed by region of residence, urban or rural setting, religion, access to healthcare, and wealth index.

Outcome and exposure variables

IPV

IPV was defined as at least one form of emotional, physical, or sexual violence from a romantic partner in the past year. IPV was categorized as a binary variable (0 = No and 1 = Yes) from variables in the dataset that measured control issues, emotional violence, physical violence, and sexual violence. Any woman that reported at least one control issue or emotional, physical, or sexual violence in the past year was categorized as having experienced IPV.

Help seeking for IPV

Help seeking for IPV, the outcome variable, was asked only of women who reported experiencing physical or sexual violence and was determined from the question, "Thinking about what you yourself have experienced among the different things we have been talking about, have you ever tried to seek help to stop the person (s) from doing this to you?" Those who answered yes were asked about the sources from where they sought help. The options were their own family, their husband or partner's family, a friend, a neighbour, a religious leader, doctor or other healthcare provider, the police, a lawyer, a social service organization, and other source not previously listed.

The socio-demographic variables were the ages of the woman and her partner, number of children the woman had, geographic region of residence in the country, place of residence (urban or rural), highest educational level of the woman and her partner (categorized as none, primary, secondary, and tertiary education), religion, wealth index of the woman (categorized as quintiles with 1 as poorest and 5 as richest), marital status, and number of other wives the woman's husband or partner had. Visit to a healthcare facility in the past 12 months was a binary variable categorized as yes or no.

The indicator variables for the LCA were the places or people from which women experiencing physical or sexual violence sought help. These were categorized as 1) their own family, 2) their

partner’s family, 3) their neighbours, 4) their friends, and 5) formal sources. The formal source category was created by collapsing help seeking from a religious leader, doctor or other healthcare provider, the police, a lawyer, or a social service organization because only 74 women sought help from these sources. Similar recategorization has been described in previous studies using DHS data.[2, 13]

Data analysis

Sampling weights were used in the analysis to account for the complex survey design and ensure that the results are representative of the target population. The weights were provided in the DHS dataset and used to adjust for unequal probabilities of selection and non-response. These sampling weights were applied to both descriptive statistics using R statistical software, using the survey package with functions *svydesign*, *svymean*, *svyquantile*, *svyby*, *svyttest*, and *svychisq*.[14] LCA using Mplus version 8.10[15] with “mixture complex” as the analysis type command was also performed. “Mixture complex” allowed for the incorporation weight, primary sampling units and strata in the analysis.

The prevalence of IPV was estimated by dividing the number of women who has at least one IPV experience in the last year by the total number women who were surveyed for this question. The prevalence of seeking help for IPV was estimated by dividing the number of women who sought help for IPV by the total number of women who experienced physical and/or sexual IPV.

For the LCA, the relative fit of the models was compared using Bayesian Information Criterion (BIC), Adjusted BIC (ABIC), Lo-Mendell-Rubin Likelihood Ratio Test (LMR) *p*-values, and entropy values. Multiple models with two to five class solutions were created. Lower BIC or ABIC values indicated a better model while the LMR *p*-values indicated if a model fit better than a model with one less class. A *p*-value of less than ·05 suggested that the model with a higher number of classes fit better. Entropy values range from zero to one, with values greater

than 0·60 indicating a good model fit.[16] Lastly, the models were assessed on clear visual demarcations and meaningfulness of the classes.

Within the selected best class model, multinomial logistic regression was conducted in a post-hoc exploratory analysis to determine the factors associated with class membership using the R3Step model in Mplus version 8.10.[17] The selected variables for this analysis were based on findings in previous studies: age of the woman, region, residence, highest level of education, wealth index, previous visit to a health facility, severity of violence, type of violence, intergenerational history of IPV, and the woman's attitudes towards IPV.[3, 5, 9]

Ethical considerations

Permission to use the publicly available dataset in this analysis was requested and granted by the demographic and health survey (DHS) program (https://dhsprogram.com/Data/). The DHS Program and the National Health Research Ethics Committee of Nigeria reviewed and approved the research protocol. Each respondent was read an informed consent statement and could verbally accept or decline to participate with no consequences. Parents or guardians provided consent for adolescents. The consent decision was witnessed and documented on the data collection forms by the interviewers. The data were only made publicly available after they were fully anonymized. The Hamilton integrated research ethics board of McMaster University further gave ethical approval for the study (Project ID: 17782).

Results

A total of 41,821 women aged 15-49 years were interviewed as part of the DHS. Of these, 10,678 women were interviewed about their experiences of domestic violence. Questions about IPV were asked of 8,910 women who were ever married or in union (Fig 1).

These 8,910 women had a mean age of 32.2 ± 11.33 years. The women had 0 to 12 children with 7% having no children and 66% having one to four children. The median number of children was three with an interquartile range of two to five. According to the survey data, 66% (3,054/8,910) of women who had ever been married or been in union reported that they had experienced at least one form of IPV.

Table 1 (complete version S1 Table in the appendix) shows that the women who experienced IPV were similar in age and number of children to those who did not. Among women who reported experiencing IPV, the women from the North West region of the country had the highest proportion (30·8%) while those the South East had the least proportion (11·3%). Women living in rural areas were more likely to report IPV (60·4%) compared with those living in urban areas (39·6%). About 40% of the women who reported IPV had no formal education. More than half of the women who reported IPV were of the Islamic religion (55·5%). The proportion of women experiencing IPV appeared to be evenly distributed across the quintiles of the wealth index and visit to a health facility in the last 12 months.

Table 1. Background characteristics of women who experienced IPV compared with those who did not (*n* = 8,910)

Characteristic	Total Study Population (*n* = 8,190)	Women who experienced IPV (*n* = 3,054)	Women who did not experience IPV (*n* = 5.136)	*p*-value
**Age (Years)&**	32.2 (11.33)	31.9 (13.53)	32.8 (19.14)	$= 4.7\times10^{-4}$
**Number of Children&**	3.5 (3.78)	3.5 (4.00)	3.4 (6.50)	$= 2.5\times10^{-1}$
**Region#**				
North Central	14.0	15.7	10.8	$< 2.2e^{-16}$
North East	15.4	18.7	9.1	
North West	29.7	30.8	27.6	
South East	11.5	11.3	12.0	
South South	10.8	12.1	8.4	
South West	18.5	11.4	32.1	
**Place of Residence#**				
Urban	43.3	39.6	50.7	$= 3.995e^{-11}$
Rural	56.7	60.4	49.3	
**Highest Educational Level#**				
No Education	41.1	43.0	37.6	$= 1.503e^{-08}$
Primary	16.6	17.2	15.3	
Secondary	32.8	32.0	34.3	
Higher	9.5	7.8	12.8	

&Mean and Standard Deviation

#Percentages (these may not add up to 100 due to rounding)

34% of women (1,041/3,054) who reported experiencing physical or sexual IPV were asked if they sought help. Figure 2 shows the distribution of the places from which they sought help. More women sought help from informal sources compared with formal sources.

Fig 2. Sources where women experiencing IPV sought help (*n* = 1,041)

*Percentages may exceed 100 as respondents were allowed to select multiple answers

Table 2 shows that the characteristics of women sought help for IPV were similar to those who did not except that woman with secondary school education sought help more than others. More women who sought help for IPV were from South East and South South regions compared to other regions.

Table 2. A comparison of the characteristics of women who sought help for IPV compared with those who did (*n* = 3,054)

Characteristic	Women who sought help for IPV (*n* = 1041)	Women who did not seek help for IPV (*n* = 2,013)	*p*-value
**Age (Years)** [&]	32.4 (10.78)	32.3 (10.92)	= 0.87
**Number of Children**[&]	3.3 (3.07)	3.4 (3.27)	= 0.79
**Region**[#]			
North Central	12.2	22.8	< 2.2e-16
North East	20.6	20.6	
North West	10.1	12.7	
South East	21.5	11.6	
South South	24.5	12.8	
South West	11.2	19.4	
**Place of Residence**[#]			= 0.16
Urban	47.8	44.1	
Rural	52.2	55.9	
**Level of Education**[#]			= 0.12
No Education	28.2	34.1	
Primary School	20.2	18.2	
Secondary School	41.6	37.7	
Higher	9.9	9.9	
**Religion**[#]			
Catholic	14.6	11.8	
Other Christian	50.7	44.1	
Islam	34.2	43.5	
Traditionalist	0.4	0.5	
Other	0.0	0.0	
**Wealth Index**[#]			= 0.07
Poorest	12.8	15.5	
Poorer	17.5	18.2	
Middle	23.0	21.3	

Richer	26.0	20.7	
Richest	20.8	24.2	
**Visit to Health Facility in Last 12 Months#**			
Yes	53.2	49.5	= 0.14
No	46.8	50.5	
**Marital Status#**			= 8.128e-06
Married	78.8	88.1	
Living with Partner	6.4	4.5	
Widowed	6.4	2.8	
Divorced	4.0	1.8	
Separated	4.4	2.8	
**Attitude Towards IPV#**			= 0.03
Non-Accepting	72.8	67.8	
Accepting	27.2	32.2	
**Intergenerational IPV History#**			= 0.0009
No	68.7	76.0	
Yes	22.7	16.1	
Don't Know	8.6	7.9	

&Mean and Standard Deviation

#Percentages (these may not add up to 100 due to rounding)

Table 3 shows that a four-class latent model best categorizes help seeking behaviour among Nigerian women who experienced physical or sexual violence and sought help for it. The five-class latent model had the lowest BIC and ABIC values (3905·90 and 3813·79), respectively. However, its entropy value (0·93) was similar to the four-class latent model (0·92) and it was not statistically different from the four-class latent model ($p = 0{\cdot}38$). Lastly, the addition of one more class to the four-class latent model did not improve the interpretability of latent classes of help seeking behaviour among these women.

Table 3. Goodness-of-fit indices comparing class membership models of seeking help for IPV

LCA Model	BIC	ABIC	LMR Comparisons of classes	*p*-value for LMR	Entropy
2-Class	4173.12	4138.18	1-Class vs 2-Class	0.0000	0.67
3-Class	4013.52	3959.53	2-Class vs 3-Class	0.0000	0.85
4-Class	3910.79	3837.74	3-Class vs 4-Class	0.0002	0.92
5-Class	3905.90	3813.79	4-Class vs 5-Class	0.38	0.93

BIC – Bayesian Information Criterion
ABIC – Adjusted Bayesian Information Criterion
LMR – Lo-Mendell-Rubin Likelihood Ratio Test

The four-class model is shown in Figure 3: Class I - Seeks Help From Own Family Alone (49·1%), Class II - Seeks Help From Everywhere (18·1%), Class III - Predominantly Seeks Help from Formal Sources (5·3%) and Class IV - Predominantly Seeks Help from Partner's Family (27·5%).

Fig 3. Latent classes of help seeking behaviour among women experiencing IPV in Nigeria (*n* = 1,041)*

*Class membership defined by a woman's probability of seeking help from each of the indicator sources

Figure 3 further shows the probability distribution of seeking help for IPV for women in each of the latent classes. The classes were named based on this probability distribution. The women belonging to latent Class I were characterised by having a 100% probability of seeking help from their own family and a 0% probability of seeking help from all other sources; therefore, this class was therefore named the Own Family Alone help seeking class. The women in latent Class II were named the Everywhere help seeking class because they had a probability greater than 0 of seeking help from each of the indicator sources: 50% probability of seeking help from their own family, 25% probability of seeking help from their partner's family, 42% from neighbour, 64% from friend, and 7% probability of seeking help from formal sources. The women in latent Class III had a 100% probability of seeking help from a formal source, only

an 8% probability of seeking help from their own family, and a 0% probability of seeking help from all other sources. As such, they were named the Predominantly Formal help seeking class. Lastly, women belonging to latent Class IV were named the Predominantly Partner Family help seeking class because their probability distribution of help seeking from the indicator classes were 100% from their partner's family, 45% from their own family, 2% from formal sources, and 0% from neighbours and friends.

Table 4 shows the factors associated with belonging to Class II, III and IV compared with Class I. It further shows the factors associated with membership within each class. Women in Class I (Own Family) were more likely to be from the North East of Nigeria than women in all the other classes: Class II (Everywhere), Class III (Predominantly Formal) and Class IV (Predominantly Partner Family). The other differences were with the women who sought help from Predominantly Formal sources (Class III); they were less likely to be from the South South of the country but more likely to have reported a previous history of sexual violence than those in Class I. Women who went everywhere for help (Class II) were more likely to have primary education as their highest level of education compared with women in Class I (Own Family).

Table 4. Factors associated with latent class membership* (*n* = 1,041)

	Odds Ratio	Standard Error	95% Confidence Interval for Odds Ratio	
**Class II: Everywhere**				
Age of Woman in Years	1.01	0.019	0.97	1.05
**Region (Reference is South West Region)**				
North Central	2.22	1.413	0.64	7.73
North East	0.15	0.085	**0.05**	**0.46**
North West	1.81	1.455	0.38	8.74
South East	0.83	0.378	0.34	2.03
South South	0.42	0.212	0.16	1.13
**Residence (Reference is Urban)**				
Rural	1.17	0.362	0.64	2.14
**Highest Level of Education of Woman (Reference is Higher Education)**				
No Education	1.75	0.921	0.62	4.91
Primary Education	2.76	1.339	**1.07**	**7.14**
Secondary Education	1.59	0.655	0.71	3.57
**Wealth Index (Reference is Richest)**				
Poorest/Poorer	1.00	0.000	1.00	1.00
Richer/Richest	1.00	0.000	1.00	1.00
Number of Children Woman Has	0.87	0.067	0.75	1.01
**Visit to a Health Facility in the Last 12 Months (Reference is Yes)**				
No Visit	1.04	0.285	0.61	1.78
**Experience of Less Severe Violence (Reference is No)**				
Yes	1.91	0.638	0.99	3.67
**Experience of Severe Violence (Reference is No)**				
Yes	1.68	0.506	0.93	3.03
**Experience of Sexual Violence (Reference is No)**				
Yes	1.84	0.704	0.86	3.89
**Intergenerational History of IPV (Reference is None)**				
Yes/Don't Know	1.00	0.00	1.00	1.00
**Woman's Attitude to IPV (Reference is Non-Accepting Attitude)**				
Accepting Attitude	1.22	0.383	0.66	2.25
**Class III: Predominantly Formal**				
Age of Woman in Years	1.01	0.033	0.94	1.07
**Region (reference is South West Region)**				
North Central	0.58	0.609	0.07	4.54
North East	0.22	0.168	**0.05**	**0.99**
North West	3.53	3.453	0.52	23.99
South East	0.26	0.220	0.05	1.37
South South	0.25	0.170	**0.06**	**0.96**
**Residence (Reference is Urban)**				
Rural Residence	1.36	0.660	0.52	3.52
**Highest Level of Education of Woman (Reference is Higher Education)**				
No Education	0.46	0.375	0.09	2.29
Primary Education	0.81	0.718	0.14	4.62
Secondary Education	0.97	0.816	0.19	5.03
**Wealth Index (Reference is Richest)**				
Poorest/Poorer	1.00	0.000	1.00	1.00
Richer/Richest	1.00	0.000	1.00	1.00
Number of Children Woman Has	0.91	0.102	0.73	1.13
**Visit to a Health Facility in the Last 12 months (Reference is Yes)**				
No Visit	0.63	0.304	0.24	1.62
**Experience of Less Severe Violence (Reference is No)**				
Yes	0.91	0.360	0.42	1.98
**Experience of Severe Violence (Reference is No)**				
Yes	2.53	1.072	1.10	5.80
**Experience of Sexual Violence (Reference is No)**				
Yes	3.12	1.522	**1.20**	**8.12**
**Intergenerational History of IPV (Reference is None)**				
Yes/Don't Know	1.00	0.000	1.00	1.00
**Woman's Attitude to IPV (Reference is Non-Accepting Attitude)**				
Accepting Attitude	1.17	0.573	0.45	3.06
**Class IV: Predominantly Partner Family**				
Age of Woman in Years	0.98	0.017	0.95	1.02
**Region (Reference is South West Region)**				
North Central	2.71	1.686	0.80	9.17
North East	0.31	0.182	**0.01**	**0.98**
North West	3.13	2.427	0.68	14.31
South East	0.46	0.241	0.16	1.29

South South	1.06	0.537	0.39	2.86
**Residence (Reference is Urban)**				
Rural Residence	1.31	0.367	0.76	2.27
**Highest Level of Education of Woman (Reference is Higher Education)**				
No Education	1.21	0.658	0.42	3.52
Primary Education	1.91	1.043	0.66	5.57
Secondary Education	1.25	0.596	0.49	3.18
**Wealth Index (Reference is Richest)**				
Poorest/Poorer	1.00	0.000	1.00	1.00
Richer/Richest	1.00	0.000	1.00	1.00
Number of Children Woman Has	0.94	0.068	0.82	1.09
**Visit to a Health Facility in the Last 12 Months (Reference is Yes)**				
No Visit	1.03	0.255	0.63	1.67
**Experience of Less Severe Violence (Reference is No)**				
Yes	0.93	0.236	0.56	1.53
**Experience of Severe Violence (Reference is No)**				
Yes	1.18	0.301	0.71	1.94
**Experience of Sexual Violence (Reference is No)**				
Yes	1.88	0.615	0.97	3.57
**Intergenerational History of IPV (Reference is None)**				
Yes/Don't Know	1.00	0.000	1.00	1.00
Woman's Attitude to IPV (Reference is Non-Accepting Attitude)				
Accepting Attitude	1.26	0.339	0.75	2.14

***** Reference Class is Class I (Own Family Only)

S2 Table showing the conditional probabilities and assigned latent classes for the women is in the appendix.

Discussion

In this study, we described an LCA of help seeking behaviour among women experiencing IPV in a resource-constrained setting. We used the most recent DHS data from Nigeria as an example of a resource-constrained setting where women rely predominantly on informal sources of help for IPV. Previous studies focused on latent classes of help seeking among women who used formal services.[9, 10]

We found that 66% of women who were ever married or had a partner had reported at least one form of emotional, physical, or sexual violence from their partner in the 12 months prior to the survey. Of these women, only 33% of those who reported physical and/or sexual violence from their partner reported that they sought help for the violence. This agrees with previous studies on help seeking for IPV among women in similar settings.[1, 2, 18, 19]

The best model for the latent classes of help seeking was a four-class model: Class I (Own Family), Class II (Everywhere), Class III (Predominantly Formal), and Class IV (Predominantly Partner Family). The covariates significantly associated with these latent classes were region of the country, previous sexual violence, and the woman's highest level of education. The latent classes of help seeking behaviour in IPV have been either two-class [20] or three-class models.[8-11] The four-class model described in this study is attributable to a delineation of family as a source of help emerging as two distinct classes—the women's own family and their partner's family.

In keeping with previous studies, 77% of the women in this survey sought help from informal sources, primarily their own family or their partner's family.[1, 13] Membership in all four classes was characterized by substantial help seeking from the women's own family (Class I – 100%, Class II – 50% and Class IV - 45%), except for women in Class III (Predominantly Formal) who were the least likely to seek help from family. Class III women had only an 8%

probability of seeking help from their own family. Concerning help seeking from a formal source, the women in Class III (Predominantly Formal) had 100% probability of seeking help from a formal source compared with the women in Class I - Own Family (0%), Class II - Everywhere (7%), and Class IV- Predominantly Partner's Family (2%).

The findings that the women in Class III (Predominantly Formal) had 100% probability of seeking help from a formal source, only 8% probability of seeking help from their own family, and 0% from other sources and a help seeking statistically associated with sexual violence is worthy of further exploration. These findings may suggest socio-cultural links between family ties or connections, sexual violence, and help seeking from formal sources.

The relatively small proportion (5%) of women in Class III (Predominantly Formal) agrees with the findings of a study in Ghana, which observed that women experiencing IPV in resource-constrained settings distrusted formal institutions as source of help.[21] It is also interesting to note that seeking help from predominantly formal sources was associated with sexual violence even though women experiencing sexual violence are the least likely to seek help.[3] This finding suggests that while women who experience sexual violence are less likely to seek help than those experiencing other forms of violence, those that do are more likely to seek help from formal as opposed to informal sources.

Contrary to other studies [3, 4, 21] severity of violence was not significantly associated with help seeking behaviour. This may be explained by the definition of severity of violence in this study as one or a combination of the actions of kicking, dragging, strangling, burning or threatening with a knife, gun, or other weapon. This reductive definition fails to acknowledge the individual, interpersonal, and socio-cultural influences on women's definition of severe IPV and their decision to seek help for it.[5] In contrast, Tenkorang et al. used a principal component analysis technique to produce a weighted summative index, which may have been

a more sensitive measure for severe violence. Similarly, in a qualitative study among women experiencing IPV in Ghana, severe violence was defined as a perception of being in danger of death.[21] This perception would be inadequately captured by the binary categorization of the listed actions in this study.

The implications of the study findings are the need for multi-faceted interventions that educate and empower both lay members of the community and workers in formal institutions to identify and respond appropriately to IPV. Interventions have often focused on formal settings such as hospitals, healthcare workers, and justice system workers but the heavy reliance on family and friends as support for women experiencing IPV suggests that community approaches to IPV prevention and response should also address these informal sources of help.[6]

This study had some limitations. We relied exclusively on secondary data from the DHS, which may not be detailed enough to capture the nuances of help seeking behaviour in a resource-constrained setting. The complexity of the process of help seeking in IPV was reduced to latent classes of behaviour constructed using binary variables developed from a pre-determined list of sources of help. There may be places, people, and resources beyond this list that can only be explored and delineated qualitatively. Nevertheless, the latent classes described in this study are important as a foundation for future studies.

In conclusion, this study has delineated the latent classes of help seeking among women experiencing IPV in a resource-constrained setting. The women evinced a high reliance on their own and partner's families for help. Women with a history of sexual violence were most likely to access formal sources of help but least likely to seek help from family. Both informal and formal systems need to be strengthened to ensure that women experiencing IPV receive the help that they need. Formal systems strengthening should specifically include sexual IPV prevention and response without neglecting other forms of IPV.

References

1. Goodson A, Hayes BE. Help-Seeking Behaviors of Intimate Partner Violence Victims: A Cross-National Analysis in Developing Nations. Journal of interpersonal violence. 2021;36(9-10):NP4705 –NP27.

2. Linos N, Slopen N, Berkman L, Subramanian SV, Kawachi I. Predictors of help-seeking behaviour among women exposed to violence in Nigeria: a multilevel analysis to evaluate the impact of contextual and individual factors. Journal of epidemiology and community health. 2014;68(3):211-7.

3. Tenkorang EY, Sedziafa AP, Owusu AY. Does Type and Severity of Violence Affect the Help-Seeking Behaviors of Victims of Intimate Partner Violence in Nigeria? Journal of Family Issues. 2017;38(14):2026 - 46.

4. Lelaurain S, Graziani P, Monaco GL. Intimate Partner Violence and Help-Seeking: A Systematic Review and Social Psychological Tracks for Future Research. European Psychologist. 2017;22(4):263-81.

5. Liang B, Goodman L, Tummala-Narra P, Weintraub S. A theoretical framework for understanding help-seeking processes among survivors of intimate partner violence. American journal of community psychology. 2005;36(1-2):71-84.

6. Bacchus LJ, Colombini M, Pearson I, Gevers A, Stöckl H, Guedes AC. Interventions that prevent or respond to intimate partner violence against women and violence against children: a systematic review. The Lancet Public Health. 2024;9(5):e326-e38.

7. National Population Commission - NPC, ICF. Nigeria Demographic and Health Survey 2018 - Final Report. Abuja, Nigeria: NPC and ICF; 2019.

8. Kaukinen C. Help-seeking strategies of female violent-crime victims: The direct and conditional effects of race and the victim-offender relationship. Journal of interpersonal violence. 2004;19:967-90.

9. Ben-Porat A. Patterns of Service Utilization Among Women Who Are Victims of Domestic Violence: The Contribution of Cultural Background, Characteristics of Violence, and Psychological Distress. Journal of interpersonal violence. 2020;35(17-18):3167-87.

10. Cheng SY, Wachter K, Kappas A, Brown ML, Messing JT, Bagwell-Gray M, et al. Patterns of Help-seeking Strategies in Response to Intimate Partner Violence: A Latent Class Analysis. Journal of interpersonal violence. 2022;37(9-10):NP6604-NP32.

11. Hanson GC, Messing JT, Anderson JC, Thaller J, Perrin NA, Glass NE. Patterns and usefulness of safety behaviors among community-based women survivors of intimate partner violence. Journal of interpersonal violence. 2021;36(17-18):8768-91.

12. Abubakar I, Dalglish SL, Angell B, Sanuade O, Abimbola S, Adamu AL, et al. The Lancet Nigeria Commission: investing in health and the future of the nation. The Lancet. 2022;399(10330):1155-200.

13. Ansara DL, Hindin MJ. Formal and informal help-seeking associated with women's and men's experiences of intimate partner violence in Canada. Social Science and Medicine. 2010;70(7):1011-8.

14. Lumley T. Survey: Analysis of Complex Survey Samples. R package version. 2023;4.

15. Muthén LK, Muthén BO. Mplus User’s Guide. 8 ed. Los Angeles, CA: Muthén & Muthén; 1998 - 2017.

16. Wang J, Wang X. Structural equation modeling: Applications using Mplus: John Wiley & Sons; 2019.

17. Asparouhov T, Muthén B. Auxiliary variables in mixture modeling: Three-step approaches using M plus. Structural equation modeling: A multidisciplinary Journal. 2014;21(3):329-41.

18. Aboagye RG, Seidu A-A, Cadri A, Salihu T, Arthur-Holmes F, Sam ST, et al. Ending violence against women: Help-seeking behaviour of women exposed to intimate partner violence in sub-Saharan Africa. PloS one. 2023;18(10):e0291913.

19. Dickson KS, Boateng EN, Adzrago D, Addo IY, Acquah E, Nyarko SH. Silent suffering: unveiling factors associated with women's inability to seek help for intimate partner violence in sub-Saharan Africa (SSA). Reproductive health. 2023;20(1):110.

20. Hu R, Xue J, Lin K, Sun IY, Wu Y, Wang X. The patterns and influencing factors of help-seeking decisions among women survivors of intimate partner violence in China. Journal of family violence. 2021;36:669-81.

21. Rohn E, Tenkorang EY. Structural and institutional barriers to help-seeking among female victims of intimate partner violence in Ghana. Journal of family violence. 2023;38(5):815-27.

Supporting information

S1 Table. Background characteristics of women who experienced IPV compared with those who did not (n = 8,910). Complete version of Table 1

S2 Table. Conditional Probabilities and Assigned Latent Classes (n=1,041)

Click here to access/download;Figure;Fig2.tif

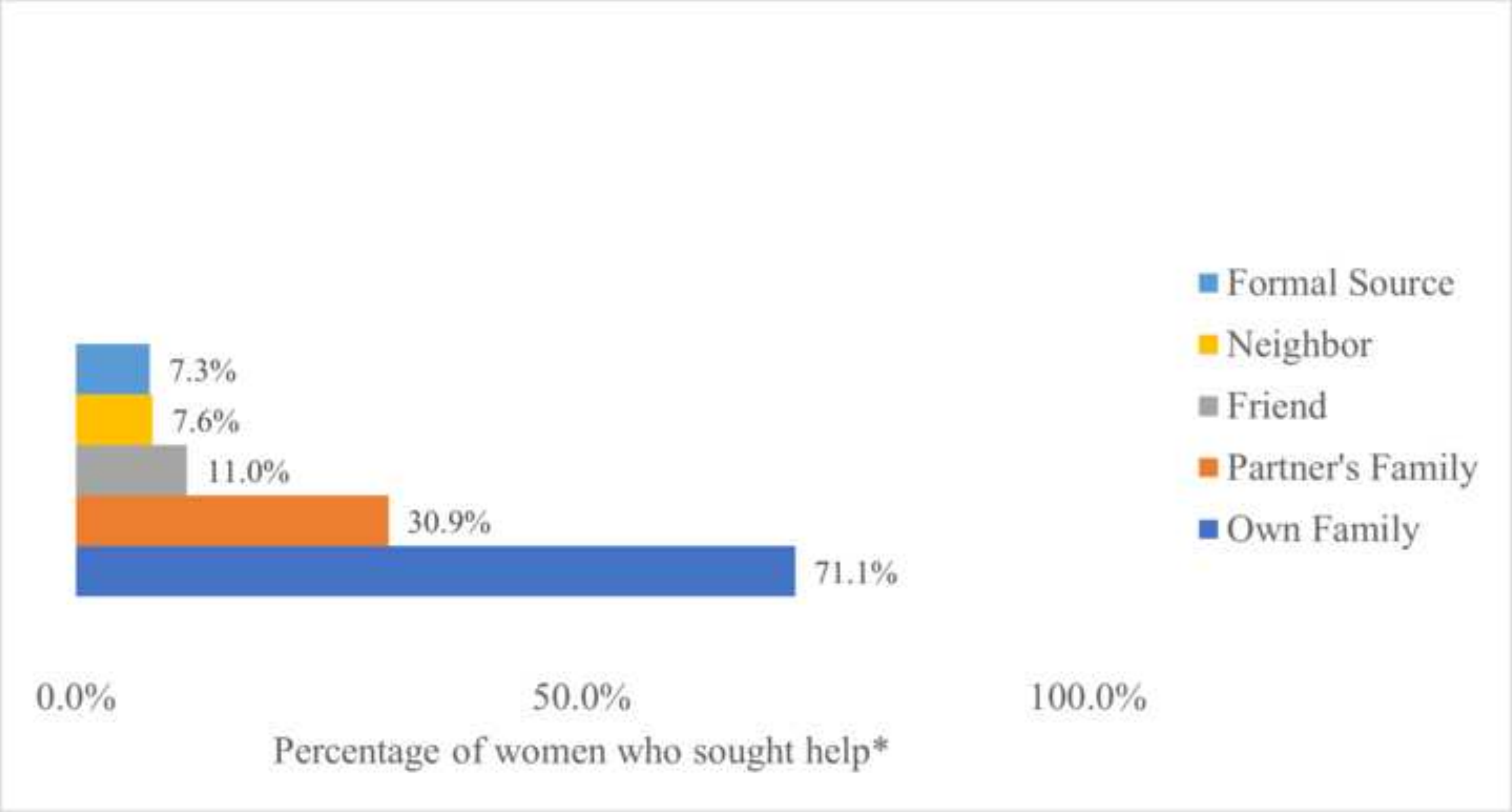

Click here to access/download;Figure;Fig3.tif

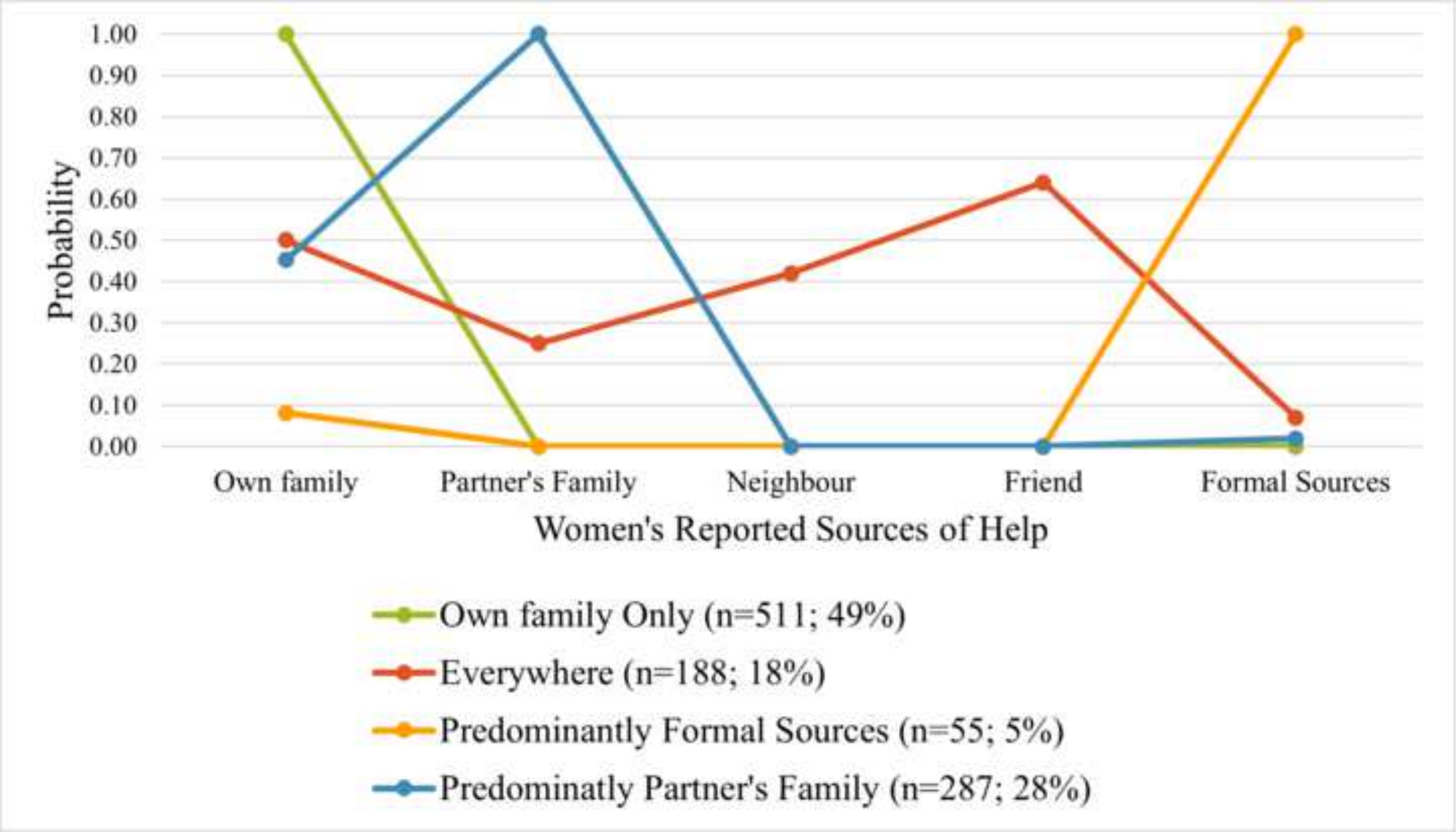

Click here to access/download
Supporting Information
S1_Table.docx

Click here to access/download
Supporting Information
S2_Table.docx

Ethics Approval

Click here to access/download
Supporting Information
Ethics_Approval_HiREB.pdf
