# Supplementary material for: Help seeking for intimate partner violence in a resource-constrained setting: A latent class analysis of the Nigerian demographic health survey dataset
Source: PLoS One. 2025 Oct 22;20(10):e0334905. doi: 10.1371/journal.pone.0334905 (PMC12543103; doi:10.1371/journal.pone.0334905)
Supplement: Attachment — Submitted filename: PONE-D-24-53009_Review.docx [file pone.0334905.s003.docx]

**PONE-D-24-53009**

**Title**: Help seeking for intimate partner violence in a resource-constrained setting: A latent class analysis of the Nigerian demographic health survey dataset

**Overall**

Authors conducted a LCA of IPV help-seeking (HS) in Nigeria. The paper has great merit, particularly in areas where little is known about available resources and support. While this paper is of great importance, it could be further strengthened by providing readers of a conceptualization of the terms, a synthesis if extant literature, identifying gaps in knowledge, better organizing the justification for this scholarship. It’s my understanding that the terms “domestic violence” is used in Nigeria rather than “intimate partner violence.” Since this is the case, I am curious how come authors did not use the terminology that is used in that country? Since authors fail to provide enough background information about IPV and underpinning dynamics for readers to contextualize the findings. Authors may also refer to the publication for organization as it is currently amiss of what is typical for academic journals. Importantly, authors discuss the 4-class model; however, the best model is actually the 5-class model, based on the BIC and entropy.

**Abstract**

1. There seems to be something missing in the significance. Need more of a justification as to why the LCA was conducted for IPV.
2. Why focus on Nigeria and not another country?
3. Why focus on help-seeking? How is it conceptualized? Authors need to make a stronger case for this.
4. The decimal is in two different places. Please ensure style is consistent for this publication.
5. What were the regions of the country?
6. What education levels?
7. Rather than previous, perhaps use the word “history” of sexual violence?
8. What are the implications of the findings?
9. What is the conclusion or takeaway for the reader? This doesn’t seem clear.

**Introduction**

1. “Devastating experience” – what is the significance of IPV victimization? What is the prevalence of IPV globally? In Nigeria? Who is impacted?
2. Do these rates differ for women in rural vs urban areas? What about the areas of the country where there is conflict?
3. Why should we care about help-seeking? Authors don’t make a case to focus on this area of research. Please convince the reader why we should care.
4. How are authors conceptualizing IPV?
5. There should really be an entire paragraph that explores the literature on help-seeking. What is it? When does it begin? Does it begin with disclosure? Is it psychological or behavioral? Or both? What does the literature say about help-seeking? What does help-seeking look like in LMICs? What about resource-constrained areas? What about help-seeking as it relates to spirituality? What theories exist on help-seeking? Waller and colleagues have developed three theories: The Theory of Help-Seeking Behavior, Constructed Agency, and Sarah Waller’s Help-Seeking Model. Do any parts of these reflect the experiences of survivors in Nigeria? How so or not? What’s missing?
6. Help-seeking varies in Nigeria…what are the underlying factors that are predictive of HS behaviors?
7. Does it matter who is the perpetrator of the physical and sexual violence as it relates to their HS behaviors?
8. What is severe IPV? How are authors conceptualizing it? Even more importantly, how did the survivors conceptualize it? Or how is it conceptualized in Nigeria?
9. Authors peppered in LMIC’s is Nigeria considered a LMIC? If so, please include how so and what is an LMIC? How is it conceptualized? Who framed it as an LMIC?
10. Graph 2 begins by stating individual, state and national factors but only explains individual. What about the state and national factors?
11. An individual strategy for HS could include relying on a range of supports from informal to formal. Since this is the case, please clarify the first sentence on graph 3.
12. Authors contradict themselves in graph 3. In the same graph author notes that survivors rely upon informal supports, but end the graph by including formal providers. Please provide more context. There are formal providers in Nigeria, but there are barriers to women obtaining such services and supports. authors may want to explain what they are and what help-seeking looks like.
13. What is missing in the literature? I am not yet convinced that this inquiry is justified although I do believe that it is.
14. Authors need to tell us more about the findings of the LCAs in the HICs rather than list that there are multiple classes.
15. What makes Nigeria a resource-constrained area? Who says it is resource constrained? Are all areas of Nigeria resource constrained? How are authors conceptualizing resource constrained?
16. HS is a process. It begins with survivors considering help-seeking and may/may not proceed with behaviors.
17. Also, US Black women are generally not reliant upon formal providers due to issues regarding individual and structural racism. They instead, largely rely upon informal networks, similar to women in Nigeria (see Waller et al, 2022).

**Methods**

1. Was data collected in-person or via telephone survey?
2. Is DHS a “program”?
3. Why would USAID commission this project? Please provide more context.
4. Where were folks recruited? Who participated?
5. Check the style for west Africa.
6. Provide folks with more background about Nigeria. What is going on with migration? Where do most folks reside?
7. What about economics? What about the wealth/income?
8. Is there conflict? If so, what areas?
9. What is the typical educational level? Do women attend school? If so, until what age? Do parents have to pay for school fees? How does this impact their educational level? What percentage of the population is educated? What level?

**Materials and methods**

1. Perhaps authors include Methods as a header that is separate. See the style for the journal.

**Sampling strategy**

1. What are EAs?
2. What was the randomization strategy?
3. Target groups … or were these participants?
4. No questions on psychological IPV? This is the most common form of IPV.
5. Was this a secondary data analysis? If so, not sure why the authors included the figure. Just state this was a SDA and provide details regarding the sampling frame, etc.
6. Unsure why authors included the info on men since the study focuses on women. Perhaps this can be deleted?

**Theoretical framework**

1. This should be before methods
2. Tell more about conceptual framework. What is it?
3. Who used it and how?
4. What are the strengths?
5. What are the limitations?

**HS for IPV**

1. Wealth index. Need more info on this—how much money do these indices represent? Is this the woman’s wealth? Her partner’s wealth? Her family or origin’s wealth?
2. What is tertiary education? Is this attending classes? Or do authors mean the person has attended university?
3. Curious how come authors did not do much with emotional (psychological violence)?

**Results**

1. Perhaps I missed this part, but what is considered formal education?
2. Perhaps authors just write the findings rather than explain in this section? For example, “evenly distributed…” This should be included in the Discussion section

**Table 1.**

1. What is the total percentage of women who have experienced IPV?
2. What types (phys, emotional/psych, sexual, financial, etc.)?

**Table 2.**

1. Are the statistically significant differences between those who sought help vs those who didn’t? What are they?

Table 3.

1. It appears the best LCA is a 5-class model rather than 4 classes based on the BIC and entropy. Curious how come authors selected the 4-class model.

**Discussion**

1. Previous studies in Nigeria focused on formal services? Perhaps I missed this. Authors cited a study on Arab women and another on US women. this is not enough justification for this study in Nigeria.
2. How do your findings relate to the extant literature? What’s new?
3. Here is the format for your discussion section. Please reorganize:
   a. Para 1: Summarize the key question/findings. Why is this an important topic, what were your main findings, why are they relevant. Eg: “In this study, we found… We also found…… Our findings are important because….” In para 1, you're telling the reader why your study matters!
   b. Para 2: Go over additional findings of importance. This paragraph should focus on secondary outcomes, those from subgroups or other sensitivity analyses that add more richness to the main findings. Eg: “In addition to our main findings, we also found that…..” Use these paras to show mastery over content. Define current state, cite key studies, describe what they've shown, what gaps remain and how do your data address these?
   c. Para 3-4: Describe whether your findings are in line with others (if so, credit them) and if not, explain why not? Eg: “Our study support prior findings by researchers X et al … However, we also found important differences including… These differences may be explained by….”
   d. Para 5-6: Limitations & strengths. Think like a reader! What may a reader think are key measurement, scientific, validity concerns? What did reviewers raise? Are there ways you have addressed these? Adding a sentence for how a limitation was managed is particularly impactful.
   e. Para 7: implications
   f. Final paras: These are key and are often missing.
      - What should future studies on this topic look like? What are the remaining questions? Does your study shed light on design or sample size or other technical issues for these studies?

- What are clinical, research or policy implications from your study? This is important and often missed! What should we do differently or be thinking about differently now that you have done this work?

-Finally, don't end with “More research is required.” More research will always be required! Instead, close with the key take home message for your paper and what you want readers to remember. EG: “Future studies that examine x,y,z accounting for a,b,c would be welcomed.”

**Conclusion**

What is the main takeaway and what do you want your readers to know. If they only read one graph, what do they need to know?

**References**

1. Authors may want to ensure they are using the latest available research (e.g. there is a 2014 citation, but there’s research conducted since then)
