# Supplementary material for: Help seeking for intimate partner violence in a resource-constrained setting: A latent class analysis of the Nigerian demographic health survey dataset
Source: PLoS One. 2025 Oct 22;20(10):e0334905. doi: 10.1371/journal.pone.0334905 (PMC12543103; doi:10.1371/journal.pone.0334905)
Supplement: Attachment — Submitted filename: Response-to-Reviewers-PONE-D-24-53009-Final.docx [file pone.0334905.s006.docx]

Mary Heersink School of Global Health and Social Medicine

Faculty of Health Sciences

McMaster University

1280 Main St. West, MDCL 3500

Hamilton, Ontario, Canada L8S 4K1

[aladeo1@mcmaster.ca](mailto:aladeo1@mcmaster.ca)

Academic Editor

PLOS ONE

**Re-submission of manuscript titled, “Help Seeking for Intimate Partner Violence in a Resource-Constrained Setting: A Latent Class Analysis of the Nigerian Demographic Health Survey Dataset- PONE-D-24-53009”**

September 30, 2025

Dear Dr. Bolarinwa,

Thank you for your consideration of the above named manuscript for publication in your journal. I appreciate the reviewers and acknowledge that their comments have helped to improve the quality and impact of this work.

Please find below a point-by-point response to each comment from the academic editor and the reviewers. I look forward to further communications regarding this manuscript.

Kind regards,

Omolola Alade

**Updated Financial Disclosure:** Not applicable.

**Academic Editor’s Comments:**

1. Please ensure that your manuscript meets PLOS ONE's style requirements, including those for file naming.

Response: This has been done.

2. We note that you have indicated that there are restrictions to data sharing for this study. PLOS only allows data to be available upon request if there are legal or ethical restrictions on sharing data publicly.

Response: The data are owned by a third-party organization who do not permit the authors to share the data. Requests for the data may be sent to https://dhsprogram.com/data/available-datasets.cfm.

**Reviewer 1 Comments:**

1. Authors conducted a LCA of IPV help-seeking (HS) in Nigeria. The paper has great merit, particularly in areas where little is known about available resources and support. While this paper is of great importance, it could be further strengthened by providing readers of a conceptualization of the terms, a synthesis if extant literature, identifying gaps in knowledge, and better organizing the justification for this scholarship.

Response: These have all been addressed in the questions below.

2. It’s my understanding that the terms “domestic violence” is used in Nigeria rather than “intimate partner violence.” Since this is the case, I am curious how come authors did not use the terminology that is used in that country? Since authors fail to provide enough background information about IPV and underpinning dynamics for readers to contextualize the findings.

Response: Domestic violence is broader than intimate partner violence and encompasses violence from any member of a household against another. Both terminologies were used in this study as explained on pages 8; lines 191 - 194 – *“In total, 10,678 women were surveyed about their experiences with domestic violence. Only 8,910 women who were ever married or partnered of the 10,678 women were asked questions on intimate partner violence (IPV).”*

On page 11; lines 230 – 235, IPV was operationalized, and this operationalization was supported with references, *“IPV was defined as at least one form of control issue, emotional, physical, or sexual violence from a romantic partner in the past year [12, 27]. IPV was categorized as a binary variable (0 = No and 1 = Yes) from variables in the dataset that measured control issues, emotional violence, physical violence, and sexual violence. Any woman that reported at least one control issue or emotional, physical, or sexual violence in the past year was categorized as having experienced IPV.”*

3. Authors may also refer to the publication for organization as it is currently amiss of what is typical for academic journals.

Response: We have followed the journal’s guidelines and have also consulted recently published articles in this journal to ensure that the organizational structure is consistent with this journal’s specifications.

4. Importantly, authors discuss the 4-class model; however, the best model is actually the 5-class model, based on the BIC and entropy.

Response: The best model was selected using a combination of statistics and subjective interpretation as supported in the literature. This has been more fully explained on pages 13 and 14; lines 301 – 309*, “For the LCA, the relative fit of the models was compared using Bayesian Information Criterion (BIC), Adjusted BIC (ABIC), Lo-Mendell-Rubin Likelihood Ratio Test (LMR) p-values, and entropy values. Multiple models with two to five class solutions were created. Lower BIC or ABIC values indicated a better model while the LMR p-values indicated if a model fit better than a model with one less class. A p-value of less than ⸱05 suggested that the model with a higher number of classes fit better. Entropy values range from zero to one, with values greater than 0⸱60 indicating a good model fit [32]. Lastly, the models were assessed on clear visual demarcations and meaningfulness of the classes [33].”*

and on page 22; lines 382 – 390, “*The five-class latent model had the lowest BIC and ABIC values (3905⸱90 and 3813⸱79), respectively. However, its entropy value (0⸱93) was similar to the four-class latent model (0⸱92) and was not statistically different from it (p = 0⸱38). Lastly, the addition of one more class to the four-class latent model did not improve the interpretability of latent classes of help seeking behaviour among these women. Therefore, the four-class latent model was selected over the five-class model, to describe the help seeking behaviour in this population, because of its parsimony, interpretability and non-statistically significant difference from (p= 0.38) from the five-class model.”*

**Abstract**

1. There seems to be something missing in the significance. Need more of a justification as to why the LCA was conducted for IPV. Why focus on Nigeria and not another country? Why focus on help-seeking? How is it conceptualized? Authors need to make a stronger case for this. The decimal is in two different places. Please ensure style is consistent for this publication. What were the regions of the country? What education levels? Rather than previous, perhaps use the word “history” of sexual violence? What are the implications of the findings? What is the conclusion or takeaway for the reader? This doesn’t seem clear.

Response: The abstract has been rewritten to address all the issues raised within the constraints of the word count limit.

**Introduction**

1. “Devastating experience” – what is the significance of IPV victimization? What is the prevalence of IPV globally? In Nigeria? Who is impacted?

Response: Thank you very much. Page 3; lines 52 – 60 now reads, *“Intimate partner violence (IPV) is a devastating experience that disproportionately affects women. Globally, about 27% of women aged 15 years and older (641 million) have been victims of IPV at least once in their lifetime [1]. When categorized by United Nations’ economic classification, the highest prevalence of lifetime experience of intimate partner violence was recorded in the Least Developed Countries (37%), while the lowest rates were observed in high income countries (16-23%) [2]. The IPV landscape in Nigeria reflects the global picture with 17 - 24% of ever married/partnered women aged 15 years and older reporting at least one exposure to IPV in their lifetime [3, 4].”*

2. Do these rates differ for women in rural vs urban areas? What about the areas of the country where there is conflict?

Response: This is outside the scope of the study which focused on help seeking for IPV rather than IPV itself. Furthermore, conflict situations in Nigeria are distinct forms of resource constraints that were not available in the data set used for this study.

3. Why should we care about help-seeking? Authors don’t make a case to focus on this area of research. Please convince the reader why we should care.

Response: We highlight that IPV is a devastating experience that women need to cope with by help seeking which has a low prevalence generally and particularly in LMICs including Nigeria. Page 3; lines 61 – 73 *“One of the ways women experiencing IPV respond is to actively reach out for assistance. This is referred to as help seeking. Help seeking is both a cognitive and behavioural process characterized by “problem focus, intentional action and interpersonal interaction with a third party” [5-7]. It is an active process that begins with the recognition of the violence situation as a problem, the realization that self-resources are inadequate to cope with the problem and the need for intervention by an external agent [5, 7, 8]. Although help seeking is an important coping strategy in IPV it is underutilized in resource constrained settings with a reported average prevalence of 35% in low- and middle-income countries (LMIC) [2]. A study in Nigeria found that only about 40% of women experiencing IPV sought help to stop the perpetrator from hurting them again [9]. The same study further found that help seeking behaviour varied widely across the states in Nigeria, ranging from 12% to 65%.”*

4. How are authors conceptualizing IPV?

Response: This has been addressed in the question on domestic violence above and in the manuscript on page 11; lines 230 – 235.

5. There should really be an entire paragraph that explores the literature on help-seeking. What is it? When does it begin? Does it begin with disclosure? Is it psychological or behavioral? Or both? What does the literature say about help-seeking? What does help-seeking look like in LMICs? What about resource-constrained areas? What about help-seeking as it relates to spirituality? What theories exist on help-seeking? Waller and colleagues have developed three theories: The Theory of Help-Seeking Behavior, Constructed Agency, and Sarah Waller’s Help-Seeking Model. Do any parts of these reflect the experiences of survivors in Nigeria? How so or not? What’s missing? Help-seeking varies in Nigeria…what are the underlying factors that are predictive of HS behaviors?

Responses: This is addressed in the Introduction and theoretical framework sections.

6. Does it matter who is the perpetrator of the physical and sexual violence as it relates to their HS behaviors? What is severe IPV? How are authors conceptualizing it? Even more importantly, how did the survivors conceptualize it? Or how is it conceptualized in Nigeria?

Response: Thank you very much. In this study, the perpetrators were all husbands/male partners of the women. Page 12; lines 270 – 277*, “A woman was categorized as having experienced less severe violence if she responded, Yes, to at least one of the following prompts: "Ever been pushed, shook or had something thrown by husband/partner", "Ever been punched with fist or hit by something harmful by husband/partner" or "Ever had arm twisted or hair pulled by husband/partner" while severe violence was recorded if a women responded, Yes, to at least one of these prompts: “Ever been kicked or dragged by husband/partner”, "Ever been strangled or burnt by husband/partner" or "Ever been threatened with knife/gun or other weapon by husband/partner" [25].”*

7. Authors peppered in LMIC’s is Nigeria considered a LMIC? If so, please include how so and what is an LMIC? How is it conceptualized? Who framed it as an LMIC?

Response: A section has been included on Resource constrained settings, Pages 5; lines 104 – 123, to address these.

8. Graph 2 begins by stating individual, state and national factors but only explains individual. What about the state and national factors?

Response: page 4; lines 76 - 79 “*State and national factors include human development indices derived from a composite of literacy rates, per capita gross domestic product, life expectancy, and legal policies on IPV such as the definition of rape and state interference with domestic issues.*”

9. An individual strategy for HS could include relying on a range of supports from informal to formal. Since this is the case, please clarify the first sentence on graph 3. Authors contradict themselves in graph 3. In the same graph author notes that survivors rely upon informal supports, but end the graph by including formal providers. Please provide more context. There are formal providers in Nigeria, but there are barriers to women obtaining such services and supports. authors may want to explain what they are and what help-seeking looks like.

Response: Page 4; lines 85 - 90 now reads as, “*Individual strategies (behaviour change, negotiating, and resisting) may be the first response to IPV but this may progress to help seeking from external sources including informal (family, friends and neighbours) and formal sources (shelters, police, legal, and medical services) [5, 11]. It is influenced by a complex interplay of individual and contextual factors including the type and severity of the IPV and may not occur due to barriers in access (financial, cultural, geographic) [11].”*

The percentages quoted in lines 94 – 97 show that less than 10% of women experiencing IPV in Nigeria seek help from a formal source, *“For example, in Nigeria, 90% of the women who sought help for IPV did so form informal sources (their own family, partner’s family, neighbours and friends) while only 7.5% of them sought help from formal institutions (healthcare institutions, legal services, social organizations, and police services) [13].”*

10. What is missing in the literature? I am not yet convinced that this inquiry is justified although I do believe that it is.

Response: We found it interesting that another reviewer stated that, “The scientific rationale for the study was explained explicitly. The methodological gap and objective of the study was clearly stated.” However, pages 4 - 5; lines 98 – 103 reads *that “The latent classes of help seeking for IPV in resource-constrained settings remains unexplored. It is important to fill this research gap and direct targeted interventions to improve help seeking in these settings [12]. Therefore, this study aimed to conduct a latent class analysis (LCA) of help seeking behaviour among women experiencing IPV using data from the most recent Nigerian Demographic Health Survey, with Nigeria being an example of a resource-constrained setting.”*

11. Authors need to tell us more about the findings of the LCAs in the HICs rather than list that there are multiple classes.

Response: This is extensively discussed in the discussion section

12. What makes Nigeria a resource-constrained area? Who says it is resource constrained? Are all areas of Nigeria resource constrained? How are authors conceptualizing resource constrained?

Response: A section has been included on Resource constrained settings, Pages 5, lines 104 – 123, to address these.

13. HS is a process. It begins with survivors considering help-seeking and may/may not proceed with behaviors. Also, US Black women are generally not reliant upon formal providers due to issues regarding individual and structural racism. They instead, largely rely upon informal networks, similar to women in Nigeria (see Waller et al, 2022)

Response: This is noted and we have addressed this in the Introduction and theoretical framework sections.

**Methods**

1. Was data collected in-person or via telephone survey? Is DHS a “program”? Why would USAID commission this project? Please provide more context. Where were folks recruited? Who participated?

Response: This section has been rewritten to provide the information requested. Page 6; lines 148 – 161. *“This was a latent class analysis (LCA) of the 2018 Nigeria Demographic Health Survey (DHS) data, the most recently available DHS survey dataset in Nigeria. The DHS program is an international collaborative effort primarily funded by the United States Agency for International Development (USAID) to conduct nationally representative health surveys in more than 90 low- and middle-income countries for about four decades [19]. It was conceived to provide high quality, comparable data across countries for the purpose of planning, monitoring, and evaluating population, health, and nutrition programs [19].*

*In Nigeria, these surveys are implemented by the National Population Commission of Nigeria and were first conducted in 1990 [13]. The survey was implemented using computer-assisted personal interviewing to collect data in person. Participants (women and men) were recruited at the household level across Nigeria and the data collection took place from 14 August to 29 December 2018. The dataset was downloaded for analysis on 16 September 2023.”*

The DHS is a program as described on its website: <https://dhsprogram.com/>

2. Check the style for west Africa. Provide folks with more background about Nigeria. What is going on with migration? Where do most folks reside? What about economics? What about the wealth/income? Is there conflict? If so, what areas? What is the typical educational level? Do women attend school? If so, until what age? Do parents have to pay for school fees? How does this impact their educational level? What percentage of the population is educated? What level?

Response: The relevant contextual information to the aim of the study and variables used for the analysis, have now been provided on pages 7 - 8; lines 162 – 178.

*“Nigeria is a country in West Africa with an estimated population of 200,000,000 people [20]. It is made up of six geo-political zones further divided into 36 states and the Federal Capital Territory, Abuja [13]. These states are further divided into Local Government Areas (LGAs) to total 774 LGAs. LGAs are further divided into enumerations areas (EAs) for a total of 662,529 EAs with an average of 856 EAs per LGA. An EA is the smallest area into the which the country is demarcated by the National Population Commission of Nigeria for census purposes [21].*

*In 2023, the World Bank estimated that 54% of the Nigerian population lived in urban areas [22]. Based on estimates in 2018, data from the World Bank show that only 3.3% of the population in Nigeria is covered by a social insurance program [23]. Concerning education, 8.1% of the population (aged 25 years or older) is estimated to have a Bachelor’s degree or equivalent with a distribution of 10.6% among males and 5.8% among females [23]. In relation to IPV, 28% of women were estimated to accept that a man was justified in beating his wife/female partner if she did at least one of 5 things – argued with him, went out without informing him, refused to have sex with him, burnt food or neglected their children [23].”*

**Materials and methods**

1. Perhaps authors include Methods as a header that is separate. See the style for the journal.

Response: We have revised the manuscript accordingly.

**Sampling strategy**

1. What are EAs?

Response: EAs are now defined on page 7; lines 168 – 169

*“An EA is the smallest area into the which the country is demarcated by the National Population Commission of Nigeria for census purposes.[6]”*

2. What was the randomization strategy?

Response: The second stage was a complete listing of households in the 1,400 EAs, out of which 41,688 households were randomly selected by computer programming (computer generated random numbers). (page 8; line 182 - 184).

3. Target groups … or were these participants?

Response: Target groups has been changed to participants (page 8; line 187).

4. No questions on psychological IPV? This is the most common form of IPV

Response: The focus of this study was on help seeking for IPV and as discussed in the methodology and in response to earlier comments, only women who reported physical and/or sexual violence were asked if they sought help for IPV.

5. Was this a secondary data analysis? If so, not sure why the authors included the figure. Just state this was a SDA and provide details regarding the sampling frame, etc.

Response: Yes, this is a secondary data analysis, the figure (borrowed from the style adopted in systematic reviews and trials) was included as a visual aid to show, in clear and simple format, why the final analysis only included 1,041 women although 41,821 women were in the survey.

6. Unsure why authors included the info on men since the study focuses on women. Perhaps this can be deleted?

Response: The information on men was included to provide the full context of the sampling strategy employed to interview women in this survey because the questions on domestic violence and intimate partner violence were asked only of women in the one third of households where men were were also interviewed.

**Theoretical framework**

1. This should be before methods. Tell more about conceptual framework. What is it? Who used it and how? What are the strengths? What are the limitations?

Response: This has been addressed in the theoretical framework section on page 6.

**HS for IPV**

1. Wealth index. Need more info on this—how much money do these indices represent? Is this the woman’s wealth? Her partner’s wealth? Her family or origin’s wealth?

Response:. This has now been detailed on pages 12; lines 261 – 269. *“The wealth index is a household level index that was calculated by principal component analysis from household characteristics such as flooring material used in the house, sanitation facilities and presence or absence of consumer goods such as refrigerators, transport vehicles in the household. Each person in the household got the same wealth score. These scores were then ranked in a distribution table and divided into quintiles. Therefore, women with a wealth index of 1 were members of a household with a wealth score in the top 20% of the wealth score distribution while women with a wealth index of 5 had household scores in the bottom 20% of the wealth score distribution [25].”*

2. What is tertiary education? Is this attending classes? Or do authors mean the person has attended university?

Response: This means completed secondary education and attending, attended or completed post-secondary education.

3. Curious how come authors did not do much with emotional (psychological violence)?

Response: The focus of this study was on help seeking for IPV and as discussed in the methodology and in response to earlier comments, only women who reported physical and/or sexual violence were asked if they sought help for IPV.

**Table 1.**

1. What is the total percentage of women who have experienced IPV? What types (phys, emotional/psych, sexual, financial, etc.)?

Response: Page 16; lines 336 – 338*. “According to the survey data, 66% (6007/8,910) of women who had ever been married or partnered reported that they had experienced at least one form of IPV.”*

**Table 2.**

1. Are the statistically significant differences between those who sought help vs those who didn’t? What are they?

Response: Page 19; lines 364 – 372, *“Table 2 shows that the characteristics of women who sought help for IPV were similar to those who did not. However, the factors that were significantly associated with women seeking help for IPV were region of the country, marital status, woman’s attitude towards IPV and intergenerational history of IPV. More women sought help for IPV from the South East and South South regions compared to other regions,*

*married women were more likely to seek help than women in other marital situations, women who were non-accepting of IPV were more likely to seek help than those who were accepting of IPV and women with no intergenerational history of IPV were more likely to seek help than those with an intergenerational history of IPV.”*

**Table 3.**

1. It appears the best LCA is a 5-class model rather than 4 classes based on the BIC and entropy. Curious how come authors selected the 4-class model.

Response: This has been addressed in an earlier comment.

**Discussion**

1. Previous studies in Nigeria focused on formal services? Perhaps I missed this. Authors cited a study on Arab women and another on US women. this is not enough justification for this study in Nigeria. How do your findings relate to the extant literature? What’s new?
2. Here is the format for your discussion section. Please reorganize:
a. Para 1: Summarize the key question/findings. Why is this an important topic, what were your main findings, why are they relevant. Eg: “In this study, we found… We also found…… Our findings are important because….” In para 1, you're telling the reader why your study matters!
b. Para 2: Go over additional findings of importance. This paragraph should focus on secondary outcomes, those from subgroups or other sensitivity analyses that add more richness to the main findings. Eg: “In addition to our main findings, we also found that…..” Use these paras to show mastery over content. Define current state, cite key studies, describe what they've shown, what gaps remain and how do your data address these?
c. Para 3-4: Describe whether your findings are in line with others (if so, credit them) and if not, explain why not? Eg: “Our study support prior findings by researchers X et al … However, we also found important differences including… These differences may be explained by….”
d. Para 5-6: Limitations & strengths. Think like a reader! What may a reader think are key measurement, scientific, validity concerns? What did reviewers raise? Are there ways you have addressed these? Adding a sentence for how a limitation was managed is particularly impactful.
e. Para 7: implications
f. Final paras: These are key and are often missing.

- What should future studies on this topic look like? What are the remaining questions? Does your study shed light on design or sample size or other technical issues for these studies?- What are clinical, research or policy implications from your study? This is important and often missed! What should we do differently or be thinking about differently now that you have done this work?

-Finally, don't end with “More research is required.” More research will always be required! Instead, close with the key take home message for your paper and what you want readers to remember. EG: “Future studies that examine x,y,z accounting for a,b,c would be welcomed.”

Conclusion. What is the main takeaway and what do you want your readers to know. If they only read one graph, what do they need to know?

Response: The discussion section has been rewritten to address the above concerns.

**References**

1. Authors may want to ensure they are using the latest available research (e.g. there is a 2014 citation, but there’s research conducted since then)

Response: The literature referenced includes research conducted in Nigeria in 2021 (Ref. No 2), 2018 (Ref No 3), 2017 (Ref. No 10), 2024 (Ref. No 12).

**Reviewer 2 Comments:**

1. The study provides relevant and timely insights into the underlying drivers of IPV. The authors provided an extensive review of existing evidence and statistically modelled the level of class membership with multiple models which further enriched the research. The goal of these types of studies is to translate research to practice by using evidence to inform policy, strategies and initiative to reduce IPV risks and improve intervention outcomes. It would be great for the authors to consider aligning the LCA with existing models such as the Socioecological model which attempts to align critical determinants at different levels of engagement. Other alternative models could be considered.

Response: The study was based on the theoretical framework of help seeking behaviour in IPV described by Liang et al. coupled with the socio-ecological model outlining individual, interpersonal, and socio-cultural influences on women’s help seeking for IPV.

2. Furthermore, the use of "help' was applied loosely and in some cases interchangeably with 'reporting IPV'. There is need to unpack the meaning of 'help' - is it only talking about IPV to someone or does it go beyond this to include counselling, protective & legal support, social protection, healthcare services, etc.

Response: Page 3; lines 61 – 67 states that, “*One of the ways women experiencing IPV respond is to actively reach out for assistance. This is referred to as help seeking. Help seeking is both a cognitive and behavioural process characterized by “problem focus, intentional action and interpersonal interaction with a third party” [5-7]. It is an active process that begins with the recognition of the violence situation as a problem, the realization that self-resources are inadequate to cope with the problem and the need for intervention by an external agent [5, 7, 8].”*

Page 11; lines 237 – 245 also state that, *“Help seeking for IPV, the outcome variable, was asked only of women who reported experiencing physical and/or sexual violence and was determined from the question, “Thinking about what you yourself have experienced among the different things we have been talking about, have you ever tried to seek help to stop the person (s) from doing this to you?” Those who answered yes were asked about the sources or people from where/whom they sought help. The options were their own family, their husband or partner’s family, a friend, a neighbour, a religious leader, doctor or other healthcare provider, the police, a lawyer, a social service organization, and other source not previously listed.”*

3. There is need to also explore the reason why IPV help is not sought (perhaps this is beyond the scope of this literature). But will be helpful in closing the loop.

Response:. This is a valid observation. However, this was a secondary analysis of an already existing dataset. The analysis was limited to available data which did not include reasons why help was not sought.

**Reviewer 3 Comments:**

1. The scientific rationale for the study was explained explicitly. The methodological gap and objective of the study was clearly stated. The design, setting, variable for the study, size and sampling techniques were outlined. Kindly state any efforts to mitigate bias.

Response: Thank you very much. This is addressed in the discussion section.

2. The result and discussion were summarised. Kindly state the limitation of the study in a sub-heading of it own.

Response: This has been revised.

3. State the conclusion as a sub-heading of it own and ensure it is comprehensive.

Response: This has been revised.

4. State the implication of the study as a sub-heading

Response: This has been revised.

**Other Comments in attached PDF:**

1. what does this mean? Define 'help' - counselling, protective & legal support, social protection, healthcare services, etc

Response: This statement has been rephrased in the abstract. First sentence of the abstract now states that, *“Help seeking for intimate partner violence (IPV) is a complex process that involves reaching out to an external party.”*

2. this is only one indicator of 'help'. Was this the main or only determinants in this analysis

Response: The main determinants for the latent class analysis are the people/places from whom/where the women sought help as discussed on page 13; lines 278 – 285, *“The indicator variables for the LCA were the places or people from which women experiencing physical or sexual violence sought help. These were categorized as 1) their own family, 2) their partner’s family, 3) their neighbours, 4) their friends, and 5) formal sources. The formal source category was created by collapsing help seeking from a religious leader, doctor or other healthcare provider, the police, a lawyer, or a social service organization into a single category because only 74 women sought help from these sources. Similar recategorization has been described in previous studies using DHS data [9, 26].”*

3. I am not sure why the authors chose state and national factors as a comparative grouping as ' individual factors'. An alternative classification may consider the Socioecological model which aligns with the subject matter.

Response: This was a restatement of findings from a specific study (Reference No 9).

4. do you mean 'five-class' model

Response: Page 22; lines 383 - 384 rephrased as, *“However, its entropy value (0⸱93) was similar to the four-class latent model (0⸱92) and not statistically different from it (p = 0⸱38).”*

5. this statement should be reviewed because it seems to be inferred the other way around

Response: This has been rephrased on pages 27 and 28; lines 468 -472, *“However, this class was associated with a history of sexual violence even though women experiencing sexual violence are the least likely to seek help [10]. This finding suggests that while women who experience sexual violence are less likely to seek help than those experiencing other forms of violence, those that do seek help are more likely to seek help from formal as opposed to informal sources.”*
